# Lymph Node Dissections for T3T4 Stage Penile Cancer Patients Without Preoperatively Detectable Lymph Node Metastasis Bring More Survival Benefits: A Propensity Matching Analysis

**DOI:** 10.3389/fonc.2021.712553

**Published:** 2021-09-24

**Authors:** Han Li, Yucheng Ma, Zhongyu Jian, Xi Jin, Liyuan Xiang, Hong Li, Kunjie Wang

**Affiliations:** ^1^ Department of Urology, Institute of Urology (Laboratory of Reconstructive Urology), West China Hospital, Sichuan University, Chengdu, China; ^2^ Department of Urology, Chengdu No. 5 People’s Hospital, Chengdu, China

**Keywords:** penile cancer, lymph node metastasis, lymph node dissections, propensity matching analysis, SEER

## Abstract

**Background and Aims:**

The current guidelines for the treatment of penile cancer patients with clinically non-invasive normal inguinal lymph nodes are still broad, so the purpose of this study is to determine which patients are suitable for lymph node dissection (LND).

**Methods:**

Histologically confirmed penile cancer patients (primary site labeled as C60.9-Penis) from 2004 to 2016 in the Surveillance, Epidemiology, and Results database were included in this analysis. Univariate and multivariate Cox regression analyses were applied to determine an overall estimate of LND on overall survival and cancer-specific survival. A 1:1 propensity matching analysis (PSM) was applied to enroll balanced baseline cohort, and further Kaplan–Meier (KM) survival analysis was used to get more reliable results.

**Results:**

Out of 4,458 histologically confirmed penile cancer patients with complete follow-up information, 1,052 patients were finally enrolled in this analysis. Age, pathological grade, T stage, and LND were identified as significant predictors for overall survival (OS) in the univariate Cox analysis. In the multivariate Cox regression, age, pathological grade, T stage, and LND were found significant. The same results were also found in the univariate and multivariate Cox regression analyses for cancer-specific survival (CSS). After the successful PSM, further KM analysis revealed that LND could bring significant OS and CSS benefits for T3T4 patients without lymph node metastasis.

**Conclusion:**

Lymph node dissection may bring survival benefits for penile cancer patients without preoperatively detectable lymph node metastasis, especially for T3T4 stage patients. Further randomized control trial is needed.

## Introduction

Penile cancer is a malignant disease with a high mortality rate. According to reported data, about one-third of patients with radical treatment still fail to achieve 5-year survival (1). Regional lymph node (LN) metastasis is a crucial prognostic factor for penile cancer (2). For pN0 penile cancer patients, 5-year cancer-specific survival (CSS) is about 85%–100%, but for lymph node metastasis patients, 5-year CSS is about 79%–89% for pN1, 17%–60% for pN2, and 0%–17% for pN3 (3, 4). Some previously published studies indicated that for patients with low-graded penile cancer (≤T1a), lymph node metastasis could be 0%–30%. For patients with higher graded penile cancer (≥T1b), lymph node metastasis could approach nearly 50% (5). Due to the high incidence of lymph node metastasis in penile cancer, a study has suggested that prophylactic lymph node dissection may provide survival benefits for patients with penile cancer regardless of their stage or grade (6). In the EAU guidelines of penile cancer, for patients with clinically normal inguinal lymph nodes (cN0), surveillance, invasive nodal staging, and prophylactic lymph node dissection (LND) are three main strategies; however, surveillance is only recommended in patients with pTis/pTa tumor. Invasive nodal staging is recommended because there is still no effective imaging technique that can be applied to detect micrometastasis (3).

However, previous studies have tended to include a small number of cases. Given the low incidence of penile cancer, therefore, a larger case-size study is needed to discuss the effect of preoperative prophylactic lymph node dissection for penile cancer on survival (6–10). The purpose of this study it is to figure out the effect of preoperative prophylactic LND on patient survival with the large number of penile cancer patients in the Surveillance, Epidemiology, and Results (SEER) database.

## Material and Methods

### Study Population

Histologically confirmed penile cancer patients (primary site labeled as C60.9-Penis) from 2004 to 2016 with complete follow-up information in the SEER database were included in this analysis. The exclusion criteria were as follows: 1) patients with any other cancer before penile cancer diagnosis, 2) patients with unclear age information or unclear tumor grade information, 3) patients with any identified positive N stage or M stage before surgery, 4) patients with any unclear TNM stage information, 5) patients with unclear lymph node dissection information, 6) patients with unclear follow-up information, and 7) patients who did not receive surgery.

Overall survival (OS) and penile CSS were the two main outcome events in this study, and the SEER follow-up project offered related information. In this study, LND was defined as four or more lymph nodes that were removed.

### Statistical Analysis

Based on the LND definition mentioned above, patients were classified as LND and non-LND groups. Baseline characteristic comparisons were performed as follows: *t*-test and the Mann–Whitney test were used to test for continuous variables that were normally distributed and non-normally distributed, respectively. Categorical variables were presented with the number (percentage) and tested by the chi-square test or the Fisher’s exact test. Univariate and multivariate Cox regression analyses were carried out to find significant risk factors for OS and CSS in penile cancer patients. To more objectively evaluate the effect of LND on the survival of penile cancer patients without lymphatic or distant organ metastasis, a 1:1 propensity score matching (PSM) was applied to generate a baseline balanced cohort. Standardized mean difference (SMD, |*d*|) was calculated to evaluate baseline balance (11). After PSM, Kaplan–Meier (KM) analysis was conducted between LND and non-LND groups for OS and CSS. Since there can be randomness in the PSM cohort, further 100 times PSM and consequent KM analysis were performed to obtain a complete result. Log-rank tests were used for KM analysis.

Since we do not know if patients have positive nodes before we take it out, so it is reasonable to recheck our results obtained from lymphatic metastasis-free cohort in the primary SEER penile cancer cohort in which patients with positive N stage or M stage were retained.

All statistical analyses above were achieved through R v.4.0.3 (www.r-project.org), and *rms*, *survival*, *caret*, *broom*, *survminer*, *Matching*, and *tableone* were the main R packages used in this study. All the reported *P*-values were two-sided, and significance was indicated as *P <*0.05.

## Results

### Characteristics of the Patients

Out of 4,458 patients identified in the SEER database between 2004 and 2019, 1,052 patients were finally enrolled in this analysis based on inclusion and exclusion criteria.

**Table 1** demonstrates the characteristics of included patients. One hundred forty-six (13.9%) patients received LND, and LND patients were significantly younger than non-LND patients (*P* < 0.001). Compared with non-LND patients, more high-grade patients (*P* < 0.001) and T3T4 patients (*P* < 0.001) received LND treatment. Since all the positive N and M stage patients were excluded, only a few patients receive chemotherapy (30, 2.9%) and radiation therapy (28, 2.7%). In all patients with LND, no positive lymph nodes were reported.

**Table 1:** Baseline characteristics of included patients.

Variables	Non-LND (*n* = 906)	LND (*n* = 146)	*P*
Age (years, mean ± SD)	63.4 ± 12.57	57.81 ± 13.16	<0.001
Race (*n*)			0.421
White	749 (82.7)	123 (84.2)	
Black	104 (11.5)	13 (8.9)	
Asian or Pacific Islander	40 (4.4)	7 (4.8)	
American Indian/Alaska Native	10 (1.1)	1 (0.7)	
Unknown	3 (0.3)	2 (1.3)	
Grade (*n*)			<0.001
Well differentiated, grade I	352 (38.9)	28 (19.2)	
Moderately differentiated, grade II	423 (46.7)	94 (64.4)	
Poorly differentiated, grade III	127 (14.0)	24 (16.4)	
Undifferentiated, grade IV	4 (0.4)	9 (6.2)	
T stage			<0.001
TaTx	4 (0.4)	0 (0.0)	
T1T2	794 (87.6)	109 (74.7)	
T3T4	108 (11.9)	37 (25.3)	
Pathological type			0.691
Squamous cell carcinoma	902 (99.6)	145 (99.3)	
Other type	4 (0.4)	1 (0.7)	
Chemotherapy (*n*)	27 (3.0)	3 (2.1)	0.533
Radiation therapy (*n*)	23 (2.5)	5 (3.4)	0.537
Regional nodes positive	/	0 (0)	/

### Univariate and Multivariate Cox Regression

**Table 2** demonstrates the univariate and multivariate for OS in penile cancer patients. In the univariate analysis stage, age (<0.001), pathological grade (grade I as the reference, grade II *P* < 0.001, grade III *P* < 0.001), and LND were significant (*P* < 0.001), but T stage (T1T2 as the reference, T3T4 *P* = 0.54) was not significant. However, T stage was identified as a significant factor (HR: 1.47, *P* = 0.007) for OS in the multivariate analysis. Similar results could be found in the Cox regression for CSS (**Table 3**). LND was a significant predictive factor for penile cancer CSS (HR = 0.42, *P* = 0.005) in the univariate analysis, and it also could be identified as a predictive factor for CSS (HR: 0.32, *P* < 0.001) after the adjustment (**Table 3**).

**Table 2:** Univariate and multivariate Cox regression for overall survival.

	Univariate analysis			Multivariate analysis		
	HR	95% CI	*P*	Adjusted HR	95% CI	*P*
Age (per year old)	1.05	(1.04, 1.06)	<0.001	1.05	(1.04, 1.06)	<0.001
Grade						
Well differentiated, grade I	Ref.			Ref.		
Moderately differentiated, grade II	1.59	(1.25, 2.01)	<0.001	1.64	(1.29, 2.09)	<0.001
Poorly differentiated, grade III	1.90	(1.39, 2.59)	<0.001	1.77	(1.29, 2.43	<0.001
Undifferentiated, grade IV^a^	/	/	/	/	/	/
T stage						
T1T2	Ref.			Ref.		
T3T4	1.31	(0.99, 1.73)	0.54	1.47	(1.11, 1.94)	0.007
Pathological type			0.36			0.39
Squamous cell carcinoma	2.50	(0.35, 17.83)		2.38	(0.33, 17.07)	
Other type	Ref.			Ref.		
Lymph node dissection (yes)	0.41	(0.27, 0.61)	<0.001	0.42	(0.28, 0.63)	<0.001
Chemotherapy (yes)	0.60	(0.35, 1.02)	0.58	0.64	(0.34, 1.14)	0.131
Radiation therapy (yes)	1.23	(0.71, 2.15)	0.457	1.14	(0.63, 2.08)	0.664

a Insufficient endpoint event for univariate or multivariate analysis.

**Table 3:** Univariate and multivariate Cox regression for cancer-specific survival.

	Univariate analysis			Multivariate analysis		
	HR	95% CI	*P*	Adjusted HR	95% CI	*P*
Age (per year old)	1.02	(1.00, 1.03)	0.01	1.01	(1.00, 1.03)	0.049
Grade						
Well differentiated, grade I	Ref.			Ref.		
Moderately differentiated, grade II	3.34	(2.16, 5.18)	<0.001	3.51	(2.26, 5.44)	<0.001
Poorly differentiated, grade III	3.38	(1.97, 5.79)	<0.001	3.24	(1.88, 5.59)	<0.001
Undifferentiated, grade IV	4.11	(0.56, 30.31)	0.166	4.56	(0.62, 33.75)	0.137
T stage						
T1T2	Ref.			Ref.		
T3T4	1.81	(1.23, 2.66)	0.002	1.84	(1.25, 2.73)	0.002
Pathological type						
Squamous cell carcinoma^a^	/	/	/	/	/	/
Other type	Ref.			Ref.		
Lymph node dissection (yes)	0.42	(0.23, 0.77)	0.005	0.32	(0.17, 0.60)	<0.001
Chemotherapy (yes)	2.05	(1.01, 4.19)	0.048	1.63	(0.76, 3.51)	0.211
Radiation therapy (yes)	1.66	(0.78, 3.55)	0.188	1.38	(0.61, 3.11)	0.434

a Insufficient endpoint event for univariate or multivariate analysis.

### Propensity Score Matching and Further KM Analysis

**Figure 1:**
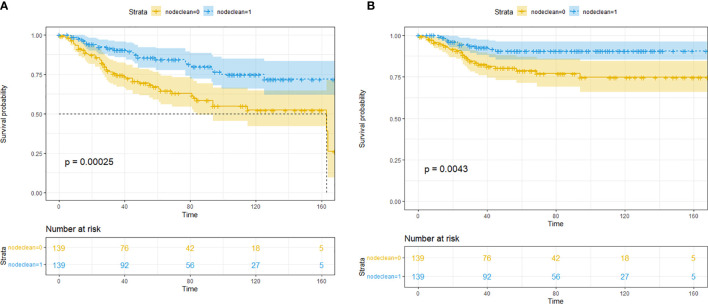
Kaplan–Meier survival analysis for lymph node dissection (LND) in the propensity score matching (PSM) cohort. **(A)** Overall survival. **(B)** Cancer-specific survival.

After the PSM, out of 86 LND patients, 139 patients were matched to 139 non-LND patients, and a total of 278 patients were enrolled into consequent KM analysis. Before the PSM, there were potential baseline differences found in age (|*d*| = 0.436), race (|*d*| = 0.148), grade (|*d*| = 0.463), and T stage (|*d*| = 0.414) between LND and non-LND patients according to |*d*| values. After the PSM, most potential baseline differences were well balanced (**Table 4**). In the KM analysis conducted within the PSM cohort (*n* = 162), LND could offer better OS (*P* = 0.00025) and CSS (*P* = 0.0043) (**Figure 1**). The main PSM cohort was generated with random seed 202104. To avoid selection bias caused by the randomness of the PSM, further 100 times PSM without random seed and consequent KM analysis were performed, and the results indicated that the main PSM results were robust for OS (*P* = 0.0025, 95% CI: 0.0014–0.0036, **Figure S3A**) and CSS (*P* = 0.024, 95% CI: 0.018–0.030, **Figure S3B**).

**Table 4:** Comparison of clinical patient characteristics between LND and non-LND groups before and after propensity score matching.

Parameters	Before propensity matching (*n* = 1,051)				After propensity matching (*n* = 1,278)			
	Non-LND (*n* = 906)	LND patients (*n* = 146)	*P*	|*d*|	LND patients (*n* = 139)	Non-LND patients (*n* = 139)	*P*	|d|
Age (mean ± SD)	63.4 ± 12.57	57.81 ± 13.16	<0.001	0.436	58.48 ± 12.86	59.02 ± 12.27	0.721	0.043
Race (*n*, %)			0.421	0.148			0.800	0.154
White	749 (82.7)	123 (84.2)			122 (87.8)	117 (84.2)		
Black	104 (11.5)	13 (8.9)			10 (7.2)	13 (9.4)		
Asian or Pacific Islander	40 (4.4)	7 (4.8)			4 (2.9)	7 (5.0)		
American Indian/Alaska Native	10 (1.1)	1 (0.7)			2 (1.4)	1 (0.7)		
Unknown	3 (0.3)	2 (1.3)			1 (0.7)	1 (0.7)		
Grade (*n*, %)			<0.001	0.463			0.947	0.040
Well differentiated, grade I	352 (38.9)	28 (19.2)			28 (20.1)	28 (20.1)		
Moderately differentiated, grade II	423 (46.7)	94 (64.4)			89 (64.0)	87 (62.6)		
Poorly differentiated, grade III	127 (14.0)	24 (16.4)			22 (15.8)	24 (17.3)		
Undifferentiated, grade IV	4 (0.4)	9 (6.2)			0 (0.0)	0 (0.0)		
T stage (*n*, %)			<0.001	0.414			0.778	0.034
TaTx	4 (0.4)	0 (0.0)			0 (0.0)	0 (0.0)		
T1T2	794 (87.6)	109 (74.7)			107 (77.0)	105 (75.5)		
T3T4	108 (11.9)	37 (25.3)			32 (23.0)	34 (24.5)		
Pathological type			0.691	0.032			1.000	0.120
Squamous cell carcinoma	902 (99.6)	145 (99.3)			139 (100.0)	138 (99.3)		
Other type	4 (0.4)	1 (0.7)			0 (0.0)	0 (0.7)		
Chemotherapy (*n*, %)	27 (3.0)	3 (2.1)	0.533	0.059	1 (0.7)	3 (2.2)	0.615	0.121
Radiation therapy (*n*, %)	23 (2.5)	5 (3.4)	0.537	0.052	2 (1.4)	5 (3.6)	0.444	0.138

**Figure 2:**
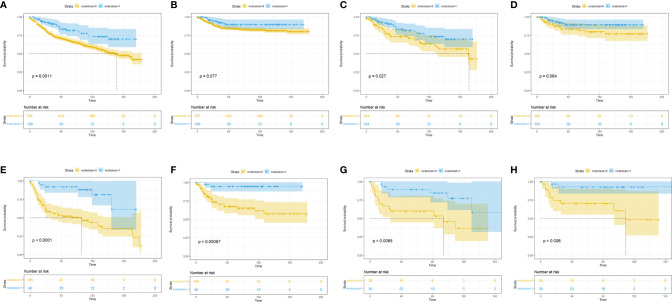
Subgroup Kaplan–Meier survival analysis for LND. **(A)** Overall survival in the T1T2 subgroup based on the full cohort. **(B)** Cancer-specific survival in the T1T2 subgroup based on the full cohort. **(C)** Overall survival in the T1T2 subgroup based on the PSM cohort. **(D)** Cancer-specific survival in the T1T2 subgroup based on the PSM cohort. **(E)** Overall survival in the T3T4 subgroup based on the full cohort. **(F)** Cancer-specific survival in the T3T4 subgroup based on the full cohort. **(G)** Overall survival in the T3T4 subgroup based on the PSM cohort. **(H)** Cancer-specific survival in the T3T4 subgroup based on the PSM cohort.

**Figure 3:**
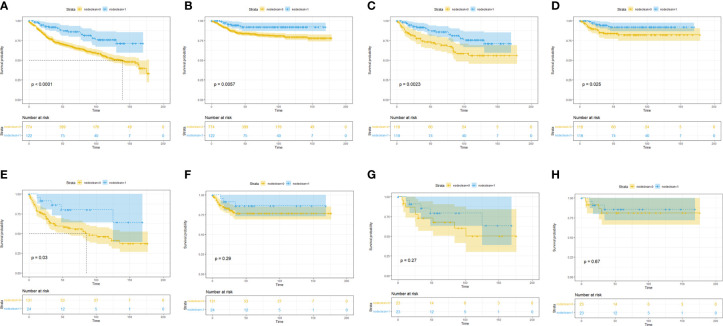
Subgroup Kaplan–Meier survival analysis for LND. **(A)** Overall survival in the grade 1/2 subgroup based on the full cohort. **(B)** Cancer-specific survival in the grade 1/2 subgroup based on the full cohort. **(C)** Overall survival in the grade 1/2 subgroup based on the PSM cohort. **(D)** Cancer-specific survival in the grade 1/2 subgroup based on the PSM cohort. **(E)** Overall survival in the grade 3/4 subgroup based on the full cohort. **(F)** Cancer-specific survival in the grade 3/4 subgroup based on the full cohort. **(G)** Overall survival in the grade 3/4 subgroup based on the PSM cohort. **(H)** Cancer-specific survival in the grade 3/4 subgroup based on the PSM cohort.

To clarify which T stage and tumor pathological grade patients could benefit from LND treatment, subgroup KM analysis was conducted. In the T stage subgroup analysis, it was found that no OS benefit could be obtained from LND for T1T2 patients, but CSS benefit could not be achieved (**Figures 2A–D**). T3T4 patients could benefit from LND for both OS and CSS (**Figures 2E–H**). In the pathological tumor grade subgroup analysis, it was found that grade 1/2 patients might obtain OS and CSS benefit from LND treatment according to the PSM results (**Figures 3A–D**), and grade 3/4 patients could not obtain OS or CSS benefit from LND (**Figures 3E–H**). However, there were only 40 T3T4 penile cancer patients analyzed in this study, the sample size was small, and related results should be treated with caution.

### Subgroup Analysis Based on the Combination of T Stage and G Stage

**Figure 4:**
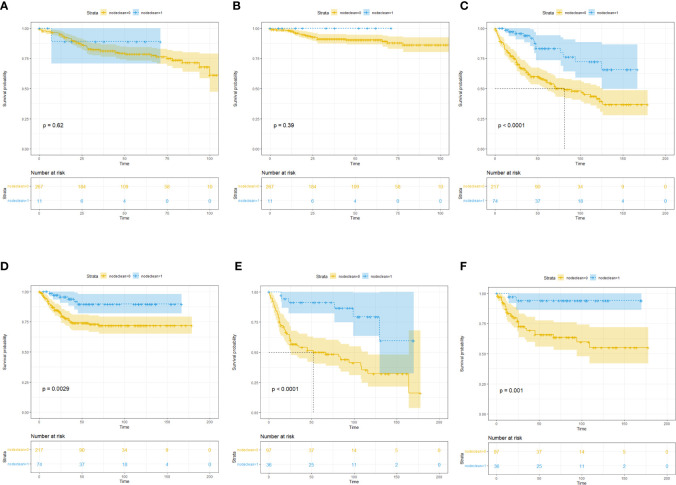
Subgroup Kaplan–Meier survival analysis for LND. **(A)** Overall survival in the Ta, T1a (G1, G2) subgroup. **(B)** Cancer-specific survival in the Ta, T1a (G1, G2) subgroup. **(C)** Overall survival in the T1b (G3) and T2 subgroup. **(D)** Cancer-specific survival in the T1b (G3) and T2 subgroup. **(E)** Overall survival in the T3 (any G) subgroup. **(F)** Cancer-specific survival in the T3 (any G) subgroup.

We further divided patients with penile cancer into Ta, T1a (G1, G2) *vs.* T1b (G3) and T2 *vs.* T3 (any G) *vs.* T4 groups to evaluate the benefit of LND in each subgroup. Considering the small number of patients in each subgroup, we did not conduct multivariate analysis and further PSM analysis. In the KM analysis, we found that in the Ta, T1a (G1, G2) group, LND could not offer OS (**Figure 4A**) or CSS (**Figure 4B**) benefits for penile cancer. This may be due to the small number of LND patients in this group, and the results were not robust. In the T1b (G3) and T2 group, LND could offer both significant OS (**Figure 4C**) and CSS (**Figure 4D**) benefits, and the same results could be also detected in the T3 (any G) group (**Figures 4E, F** **)**. This phenomenon may indicate that the lower the degree of differentiation, the higher the possibility of metastasis for penile cancer cells. However, since there were only 12 patients in the T4 subgroup, KM analysis was omitted.

### Validation in the Primary SEER Penile Cancer Cohort

The above analysis was based on a cohort of patients with non-lymph node metastatic penile cancer confirmed by preoperative physical examination, imaging examination, and postoperative pathology (although micrometastases are still possible). However, in clinical practice, it is difficult to confirm the status of lymphatic metastases prior to lymph node biopsy or LND. Therefore, it is necessary to validate the above results in the original SEER database cohort without excluding the positive N stage patients.

**Figure 5:**
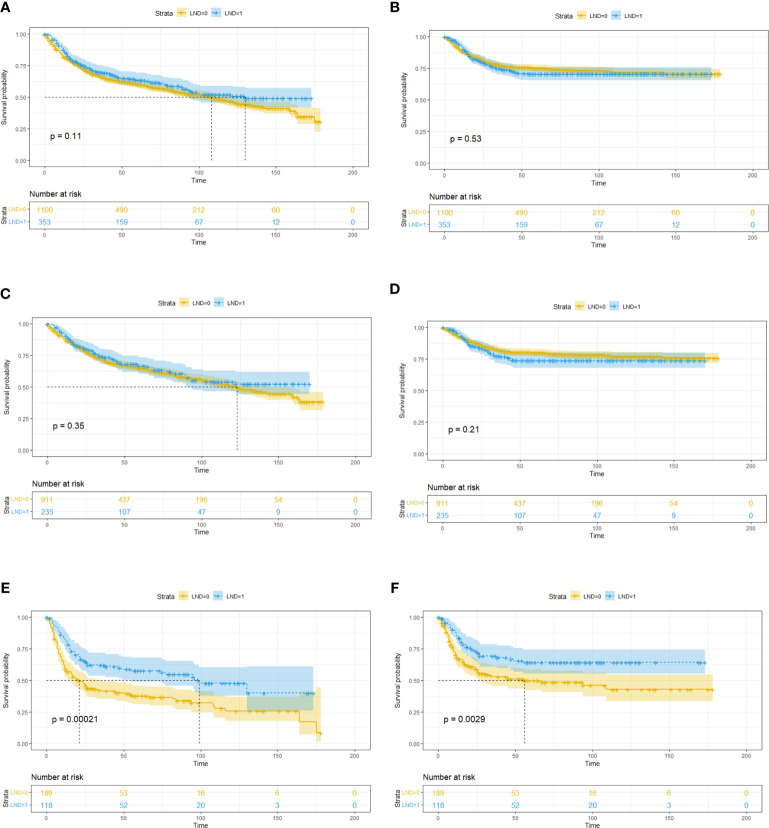
Subgroup Kaplan–Meier survival analysis for LND in the primary SEER penile cancer cohort (patients with positive N stage or M stage were retained). **(A)** Overall survival for the whole cohort. **(B)** Cancer-specific survival for the whole cohort. **(C)** Overall survival in the T1T2 subgroup. **(D)** Cancer-specific survival in the T1T2 subgroup. **(E)** Overall survival in the T3T4 subgroup. **(F)** Cancer-specific survival in the T3T4 subgroup.

Baseline comparisons for the primary SEER penile cancer cohort are shown in **Table S1**. In the KM analysis for the full cohort, T1T2 subgroup, and T3T4 group, LND could only bring OS and CSS benefits in the T3T4 subgroup, which was consistent with previous conclusions (**Figure 5**). In the further multivariate Cox regression analysis, LND was still a significant predictive factor for T3T4 penile cancer patients (**Table 5**), which was also robust.

**Table 5:** Multivariate Cox regression analysis for LND in the T3T4 subgroup penile cancer patients.

Clinical variable	Multivariate Cox regression		
	Adjusted HR*	95% CI	*P*
LND	0.51 (for OS)	(0.37, 0.72)	<0.001
	0.48 (for CSS)	(0.32, 0.72)	<0.001

*HR was adjusted by age, tumor grades, T stages, pathological type, chemotherapy history and radiation therapy history.

## Discussion

In this study, we found that, parallel to many previous studies, T stage and pathological grading of penile cancer are important prognostic factors (12, 13). In the univariate and multivariate analyses for OS, LND was a significant risk factor (HR: 1.81, *P* < 0.001). In the univariate and multivariate analyses for CSS, LND was a significant predictive factor (HR: 0.42, *P* = 0.034). To avoid potential selection bias and baseline imbalance bias, analysis after postrandomization procedures found that LND could offer both OS (*P* = 0.0073) and CSS (*P* = 0.0063) benefits in the PSM cohort. Further subgroup analysis indicated that LND could offer OS or CSS benefits for T3T4 patients but not for T1T2 patients. In the pathological grade subgroup analysis, grade 1/2 patients could obtain OS and CSS benefits from LND, but grade 3/4 patients could not.

Nowadays, penile cancer is a rare urinary cancer but with significant mortality (7). The primary pathological type of penile cancer is squamous cell carcinoma, and other pathological types only account for a tiny proportion of the total (14). In this study, more than 90% are squamous cell penile carcinoma (and its subtype). In developed countries, the incidence of penile cancer is very low, and this phenomenon may be related to penile cancer risk factors (15). Although no comprehensive meta-analysis of penile cancer risk factors has been published, some studies have indicated that HPV infection, circumcision, and hygiene may play a significant role (16, 17). The current surgical treatment for penile cancer includes organ-sparing therapy and radical treatment (3, 18). For non-invasive penile cancer involving only the glans, partial glansectomy and total glansectomy are the main surgical options (3). The most critical procedure of organ-sparing surgery is to ensure a negative margin (19). For invasive penile cancer, the surgical plan should be determined according to the different sites and extent of tumor invasion (20–22).

Lymph node metastases of penile carcinoma are usually carried out in anatomic order, starting with superficial or deep inguinal lymph nodes followed by pelvic lymph nodes (23, 24). Radical inguinal lymph node dissection or pelvic lymph node dissection should be recommended for patients with detectable preoperative lymph node metastasis (3, 25). For patients whose lymph node metastases cannot be detected preoperatively, the current main guidelines recommend that monitoring, lymph node biopsy, and radical lymphatic dissection are all acceptable (3, 26). However, considering the high probability of lymph node micrometastases in penile cancer patients, some studies suggest that active lymph node dissection can still benefit patients with negative lymph nodes examined preoperatively (27, 28). With the existing imaging methods, it is challenging to detect metastases in a small number of tumor cells before they form detectable tissue masses effectively. When the biopsy is used to detect lymph nodes, it is also challenging to avoid insufficient sampling. However, radical LND for penile cancer is highly associated with postoperative complications. Based on previously published studies, overall postoperative complication after the radical LND for penile cancer was about 80% including hematoma, lymphocele, skin necrosis, infection, and chronic scrotal pain, and the major complication was about 20% (29, 30). Therefore, if it is not clear that LND can indeed bring significant survival benefits, urologists always have many worries when taking LND for penile cancer.

According to the results of this study, a more aggressive lymph node dissection strategy for penile cancer patients with the higher stage (T3T4) may provide survival benefits. However, since the SEER database does not provide data about the intraoperative and postoperative complications of the patients, it is difficult to assess the impact of an aggressive lymph node dissection strategy on patients. Therefore, we suggest that when considering lymph node dissection strategies for patients with higher stages, the primary conditions of patients should also be considered to avoid complications as far as possible. At present, many valuable studies have been published on whether LND should be performed (31–33). We should make full use of existing tools to evaluate whether LND is needed.

There are still some limitations in this study. SEER is a population registry including a high percentage of patients diagnosed with penile cancer but not all of them. Second, no information on the template used for LND nor the technique are available (availability of frozen section, unilateral *vs.* bilateral, superficial *vs.* extended LND). Third, it does not include information on the performance status of the patients. This is clearly associated with the decision to perform LND or not.

## Conclusion

Lymph node dissection may bring survival benefits for penile cancer patients without preoperatively detectable lymph node metastasis, especially for T3T4 stage patients. Further randomized control trial is needed.

## Data Availability Statement

The original contributions presented in the study are included in the article/**Supplementary Material**. Further inquiries can be directed to the corresponding author.

## Author Contributions

KW had full access to all the data in the study and takes responsibility for the integrity of the data and the accuracy of the data analysis. Study concept and design: HaL, YM, and KW. Acquisition of data: YM, ZJ, HaL, and KW. Analysis and interpretation of data: HaL, YM, ZJ, XJ, and LX. Drafting of the manuscript: YM, HL, KW, and HaL. Critical revision of the manuscript for important intellectual content: YM, ZJ, HaL, and KW. Statistical analysis: YM and ZJ. Obtaining funding: KW and HaL. Administrative, technical, or material support: KW. Supervision: HoL and KW. All authors contributed to the article and approved the submitted version.

## Funding

This study was supported by the 1.3.5 Project for Disciplines of Excellence, West China Hospital, Sichuan University (ZY2016104, ZYGD18011).

## Conflict of Interest

The authors declare that the research was conducted in the absence of any commercial or financial relationships that could be construed as a potential conflict of interest.

## Publisher’s Note

All claims expressed in this article are solely those of the authors and do not necessarily represent those of their affiliated organizations, or those of the publisher, the editors and the reviewers. Any product that may be evaluated in this article, or claim that may be made by its manufacturer, is not guaranteed or endorsed by the publisher.

## Supplementary Material

Supplementary Table 1 Baseline characteristic of included patients.

Supplementary Figure 1 Schoenfeld residual test for variables included in the over-all survival Cox regression.

Supplementary Figure 2 Schoenfeld residual test for variables included in the cancer-specific survival Cox regression.

Supplementary Figure 3 P value distributions of KM analysis for over-all survival **(A)** and cancer-specific survival **(B)** in 100 PSM cohorts.
