# Supplementary material for: Lymph Node Dissections for T3T4 Stage Penile Cancer Patients Without Preoperatively Detectable Lymph Node Metastasis Bring More Survival Benefits: A Propensity Matching Analysis
Source: Front Oncol. 2021 Sep 24;11:712553. doi: 10.3389/fonc.2021.712553 (PMC8497980; doi:10.3389/fonc.2021.712553)
Supplement: Supplementary Table 1 — Baseline characteristic of included patients. [file DataSheet_1.docx]

**Supplementary figure 1. Schoenfeld residual test for variables included in the over-all survival Cox regression.**


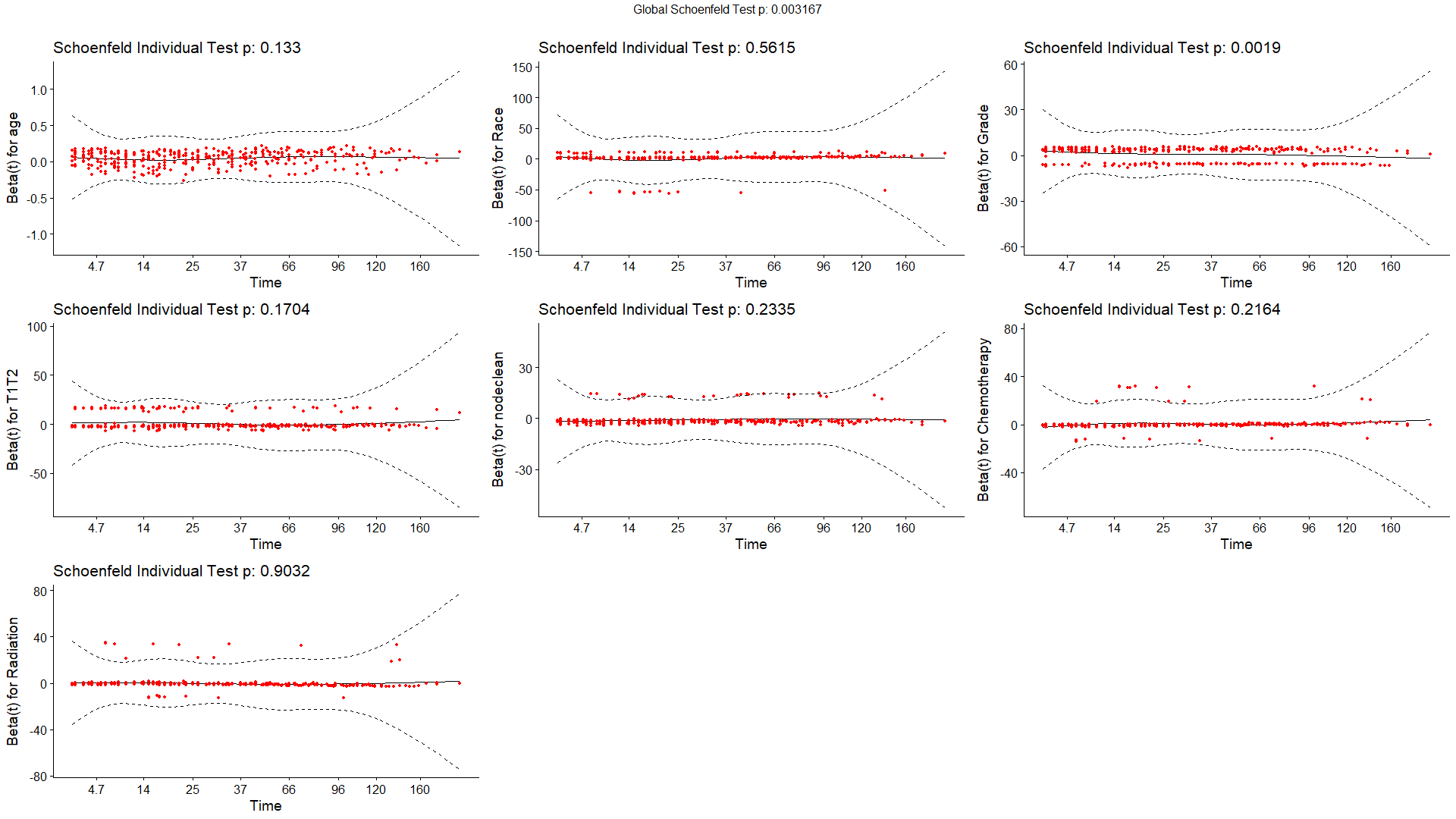


**Supplementary figure 2. Schoenfeld residual test for variables included in the cancer-specific survival Cox regression.**


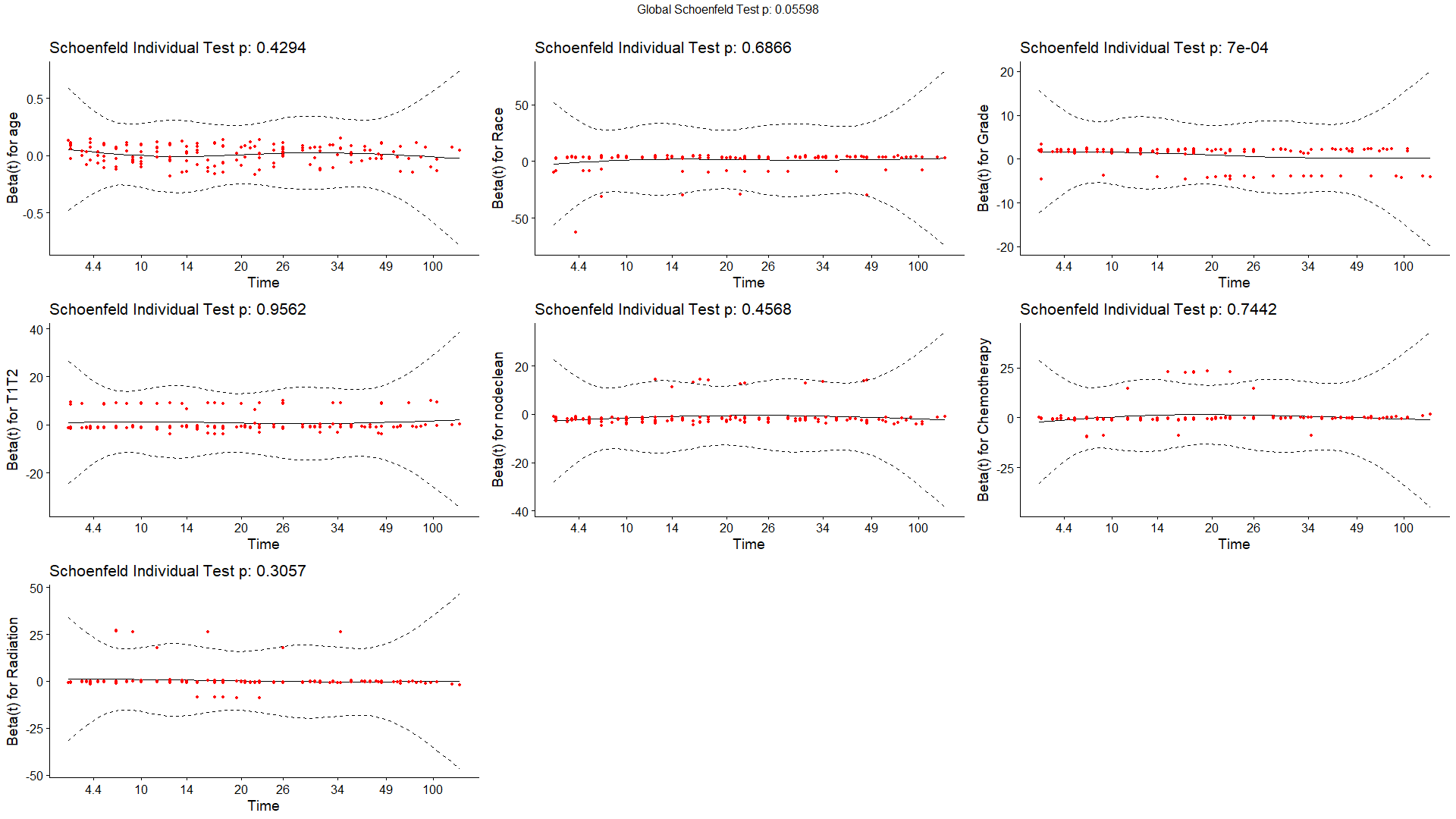


**Supplementary figure 3. P value distributions of KM analysis for over-all survival (A) and cancer-specific survival (B) in 100 PSM cohorts.**

**Table S1. Baseline characteristic of included patients**

| **Variables** |  | Non-LND (n=1100) | LND  (n=353) | P |
| --- | --- | --- | --- | --- |
| **Age (year, mean±SD)** |  | 63.3±12.52 | 59.05±12.73 | <0.001 |
| **Race (n,%)** |  |  |  | 0.58 |
| White |  | 911 (82.8) | 292 (82.7) |  |
| Black |  | 123 (11.2) | 36 (10.2) |  |
| Asian or Pacific Islander |  | 47 (4.2) | 18 (5.1) |  |
| American Indian/Alaska Native |  | 16 (1.5) | 4 (1.1) |  |
| Unknow |  | 3 (0.03) | 3 (0.8) |  |
| **Grade (n)** |  |  |  | <0.001 |
| Well differentiated, Grade I |  | 373 (33.9) | 50 (14.2) |  |
| Moderately differentiated, Grade II |  | 521 (47.4) | 210 (59.5) |  |
| Poorly differentiated; Grade III |  | 199 (18.1) | 91 (25.8) |  |
| Undifferentiated; Grade Ⅳ |  | 7 (0.1) | 2 (0.6) |  |
| **T stage** |  |  |  | <0.001 |
| TaT1T2 |  | 911 (82.8) | 235 (66.6) |  |
| T3T4 |  | 189 (17.2) | 118 (33.4) |  |
| **Pathological type** |  |  |  | 0.006 |
| Squamous cell carcinoma |  | 1035 (94,1) | 345 (97.7) |  |
| Other type |  | 65 (5.9) | 8 (2.3) |  |
| **Chemotherapy (n)** |  | 95 (8.6) | 81 (22.9) | <0.001 |
| **Radiation therapy (n)** |  | 70 (6.4) | 40 (11.3) | 0.002 |
| **Regional nodes positive** |  | 122 (11.1) | 179 (50.7) | <0.001 |
